# The role of gender inequality and health expenditure on the coverage of demand for family planning satisfied by modern contraceptives: a multilevel analysis of cross-sectional studies in 14 LAC countries

**DOI:** 10.1016/j.lana.2023.100435

**Published:** 2023-02-08

**Authors:** Laísa Rodrigues Moreira, Cauane Blumenberg, Beatriz Elena Caicedo Velasquez, Fernanda Ewerling, Alejandra Balandrán, Luis Paulo Vidaletti, Andrea Ramirez Varela, Franciele Hellwig, Rodolfo Gomez Ponce de Leon, Aluisio J.D. Barros, Mariangela Freitas Silveira, Fernando C. Wehrmeister

**Affiliations:** a Federal University of Pelotas, International Center for Equity in Health, 1160 Marechal Deodoro St., 3rd floor, Pelotas, RS, 96020220, Brazil; b Federal University of Pelotas, Post-Graduation Program in Epidemiology, 1160 Marechal Deodoro St., 3rd floor, Pelotas, RS, 96020220, Brazil; c Facultad Nacional de Salud Pública, Universidad de Antioquia, Medellín, Colombia; d Grupo de Investigación Epidemiología y Bioestadística, Universidad CES, Medellín, Colombia; e Inter-american Conference on Social Security, Direction of Health Systems Evaluation & Research, San Ramón s/n, San Jerónimo Lídice, 10200, Mexico City, Mexico; f School of Medicine, Universidad de los Andes, Bogota, Colombia; g Latin American Center for Perinatology/Women's Health and Reproductive Health of the Pan American Health Organization (CLAP/WR-PAHO/ WHO), Montevideo, Uruguay; h Institute for Global Public Health (IGPH), Rady Faculty of Health Sciences, Community Health Sciences, R070 Med Rehab Bldg, 771 McDermot Avenue, University of Manitoba (Bannatyne Campus), Winnipeg, Manitoba, R3E 0T6, Canada

**Keywords:** Family planning, Contraception, Socioeconomic factors, Multilevel analysis

## Abstract

**Background:**

Despite international efforts to improve reproductive health indicators, little attention is paid to the contributions of contextual factors to modern contraceptive coverage, especially in the Latin America and the Caribbean (LAC) region. This study aimed to identify the association between country-level Gender Inequality and Health Expenditure with demand for family planning satisfied by modern contraceptive methods (DFPSm) in Latin American sexually active women.

**Methods:**

Our analyses included data from the most recent (post-2010) Demographic and Health Survey or Multiple Indicator Cluster Survey from 14 LAC countries. Descriptive analyses and multilevel logistic regressions were performed. Six individual-level factors were included. The effect of the country-level factors Gender Inequality Index (GII) and Current Health Expenditure on DFPSm was investigated.

**Findings:**

DFPSm ranged from 41.8% (95% CI: 40.2–43.5) in Haiti to 85.6% (95% CI: 84.9–86.3) in Colombia, with an overall median coverage of 77.8%. A direct association between the odds of DFPSm and woman's education, wealth index, and the number of children was identified. Women from countries in the highest GII tertile were less likely (OR: 0.32, 95% CI: 0.13–0.76) to have DFPSm than those living in countries in the lowest tertile.

**Interpretation:**

Understanding the contribution of country-level factors to modern contraception may allow macro-level actions focused on the population's reproductive needs. In this sense, country-level gender inequalities play an important role, as well as individual factors such as wealth and education.

**Funding:**

10.13039/100000865Bill and Melinda Gates Foundation and 10.13039/501100012418Associação Brasileira de Saúde Coletiva (ABRASCO).

**Research in context**

**Evidence before this study**

We searched PubMed database using the search terms (“Contextual factors”) AND (“Family planning”) AND (“Multilevel”), with no language restrictions, for results up to November 19, 2022. Using this search strategy, we have identified only 11 studies. Most of these references were studies conducted in Africa, indicating the importance of contextual factors, with only three multi-country studies. Furthermore, in the PubMed database, 130 references were available when we used the following search strategy (“contextual factors” OR “Gender inequality” OR “Health expenditure”) AND (“Family planning”), indicating an existence of few previous research and possible gaps for this area. In the Latin America and the Caribbean (LAC) region, efforts to improve reproductive health indicators were implemented over time, leading to a rapid decline in the fertility rate in the last decades. However, there is no consensus on which factors are more relevant to the demand for family planning satisfied by modern contraceptive methods (DFPSm) at the country level.

**Added value of this study**

This study presents the first evidence that Gender Inequality Index, a country-level measure of disadvantages affecting women (dimensions: empowerment, reproductive health, and labour market), contributes to coverage of the demand for family planning satisfied. Macro and individual-level factors should be considered when analysing family planning. Especially related to gender inequality, the lower gender inequality in the country, the higher advantages to achieving universal coverage for demand for family planning satisfied with modern methods.

**Implications of all the available evidence**

International efforts to improve sexual and reproductive health indicators were implemented over time. This study provides key evidence for practice and policy, implying that including macro-level approaches focused on reducing gender disparities and considering individual-level factors is important in this field. Improving the current indicators by including contextually relevant factors to the use of modern contraception is promising.

## Introduction

In the last decades, the total fertility rate rapidly declined in the Latin America and Caribbean (LAC) region, from 5.9 births per woman in 1960 to 1.9 births per woman in 2020, producing demographic, social, and economic changes in this region, affecting the age structure and life expectancy.^1^^,^^2^ In the LAC region, there are evident characteristics such as high adolescent fertility rates (60 births per 1000 women ages 15–19 in 2020), high urban population percentages (81% of the total population in 2021), high total unemployment percentages (9.7% of the total labour force in 2021–national estimate), and a total life expectancy at birth of 76 years in 2020.^2^ The demand for family planning satisfied by modern contraceptive methods (indicator that includes women of reproductive age in need of contraception in its denominator) varies between LAC countries. Brazil (93.7%), Ecuador (89.8%), Cuba (89.5%), Costa Rica (86.8%), Colombia (83.2%), Dominican Republic (82.9%), and Mexico (81.5%) are countries with higher coverage of demand for family planning satisfied by modern contraceptive methods, while Bolivia (43.4%) and Haiti (44.1%) are identified as countries with low levels of coverage.^3^, ^4^, ^5^ In this sense, it is important to understand the processes producing these changes and the possible factors influencing the LAC indicators.

Countries in the LAC region present low national fertility rates, which vary according to population subgroups. Contraceptive use also presents inequalities, especially regarding the use of long-acting reversible contraceptives (LARCs).^3^^,^^6^ The subgroups of sexually active women from the rural area, adolescents 15–17 years old, from lower wealth quintiles, indigenous ethnicity, and with no education presented a lower prevalence of LARC contraception compared to their peers in the same country.^6^ The limited offer of a mix of modern contraceptive methods, or even the lack of availability of modern contraception and reduced access to health care, contribute to long-lasting inequalities in some localities.^7^^,^^8^ In this sense, the Sustainable Development Goals offer directions to improve family planning actions, especially targeting access and availability of contraceptive methods for all.^9^ These directions mainly target individual-level factors that are strongly associated with modern contraceptive methods utilization, including the level of education, age at first sex, marital status, and mass media exposure.^10^, ^11^, ^12^ More recently, country-level factors have also been explored as potential factors that could affect the use of modern contraceptive methods due to their possible implications on women's sexual and reproductive health, such as influences on unintended pregnancy estimates, community knowledge level of modern contraceptives and attitudes towards family planning.^10^^,^^13^, ^14^, ^15^

Recent analyses show that modern contraception is affected by contextual-level factors, including a convenient location of health facilities, exposure to family planning messages, living in localities with low maternal mortality and high antenatal care coverage, and aspects related to the quality of family planning care.^13^^,^^16^, ^17^, ^18^ However, there is no consensus on which contextual factors influence family planning coverage between LAC countries.

Worldwide, Gender Inequality Index (GII) was pointed out as an important contextual contributor to different health-related outcomes.^19^ Furthermore, GII includes empowerment as one of its dimensions. Studies have demonstrated the effects of different domains of women's empowerment on family planning.^20^^,^^21^ However, the effects of gender inequality as a contextual-level factor on family planning are not clear.

In addition, Health Expenditure and Gross Domestic Product (GDP) were mentioned as relevant contextual factors that allow worldwide comparisons for various health indicators using different analytical approaches.^22^, ^23^, ^24^ Evidence has also indicated that country-level expenditures on reproductive health and family planning contributed to the use of contraception.^25^, ^26^, ^27^ However, there is a lack of contextual data available for reproductive health and family planning expenditure in different countries, especially for those from the LAC region. In this sense, the investigation of the health expenditure seems to be a suitable approximation to the country's investments.^22^

Investigations about the use of contraception and its associated factors are available in the scientific literature, but multilevel analyses assessing simultaneously the influence of country-level factors and individual factors on demand for family planning satisfied by modern contraceptive methods are scarce, particularly in the LAC region.^10^, ^11^, ^12^^,^^14^^,^^15^^,^^28^ Ignoring the intertwined effects of these factors could mask inter-country, regional, and inter-regional reproductive health differences worldwide. This study aimed to investigate individual and country-level factors' roles in the demand for family planning satisfied by modern contraceptive methods (DFPSm). In particular, this research seeks to examine whether country-level GII, and Health Expenditure affect DFPSm above and beyond women's individual-level characteristics.

## Methods

We investigated data of 109,149 sexually active women of reproductive age (15–49 years old), irrespective of marital status, from 14 LAC countries: Belize, Colombia, Costa Rica, Cuba, Dominican Republic, El Salvador, Guatemala, Guyana, Haiti, Honduras, Mexico, Paraguay, Suriname, and Trinidad and Tobago. For each country, the most recent (post-2010) Multiple Indicator Cluster Surveys (MICS) or Demographic and Health Surveys (DHS) were included in the analyses. DHS and MICS are publicly available population-based cross-sectional standardised surveys that allow the comparison of indicators between countries.^29^, ^30^, ^31^ Both surveys aimed to investigate child, maternal, and reproductive health data using design peculiarities and multistage sampling strategies for participants' selection, with more detailed information available in the Supplementary Material (File 1) and elsewhere.^29^, ^30^, ^31^ Data has a natural hierarchy structure with 109,149 women nested within the 14 countries. Other studies using DHS and MICS surveys for epidemiological or public health research were also identified in the LAC context.^32^, ^33^, ^34^, ^35^, ^36^, ^37^

### Sources of data

Data about the DHS and MICS cross-sectional surveys were obtained from:

<https://dhsprogram.com/data/available-datasets.cfm> and <https://mics.unicef.org/surveys>, respectively. Data for GDP per capita were available at the World Bank website data <https://data.worldbank.org/indicator/NY.GDP.PCAP.CD?view=chart>. The CHE%GDP data were accessed in the Global Health Expenditure Database <https://apps.who.int/nha/database/Select/Indicators/en> with data available up to 2019. We used country-level data corresponding to the survey year included in the analysis. For the Gender Inequality Index (GII), data were available at Human Development Data Center <http://hdr.undp.org/en/data>.

### Outcome definition

DFPSm was defined as among women of reproductive age (15–49 years old), sexually active at the moment of the interview (married or in a union; or women who had sexual intercourse in the last 30 days), and in need of contraception, those who are using modern contraceptive methods.

Modern contraceptive methods included male and female sterilisation, subdermal implants, intrauterine devices, oral contraceptives, male and female condoms, emergency contraceptive pills, injectables, vaginal rings, and patches.^38^ Women were considered in need of family planning if they were fecund and did not intend to become pregnant within the next two years or were unsure about when or whether they wanted to become pregnant.^30^ In addition, pregnant women whose pregnancy was mistimed or unwanted were also defined as needing contraceptive use. This indicator better captures the success and gaps in family planning programs. It illustrates a strong commitment to the rights of individuals and couples to determine the number and timing of their children.^39^ Also, this indicator is part of those monitored by the Sustainable development goals.

### Independent factors

Independent factors were grouped into individual- and country-level factors.

The choice of the individual-level independent factors to be included was motivated by the scientific literature in this area.^10^, ^11^, ^12^, ^13^, ^14^, ^15^^,^^28^ Individual-level factors included: 1) marital status; 2) current woman's age; 3) woman's schooling; 4) wealth index (constructed based on a principal component analysis including household characteristics and ownership of selected assets)^40^; 5) area of residence; and 6) number of children.

Regarding the country-level independent factors, in this study, we decided to include more simple and general known factors to be cautious, avoiding a possible black box effect and any spurious relationships. Country-level factors were: Current Health Expenditure (CHE) and Gender Inequality Index (GII). The CHE was measured by multiplying the CHE as a percentage (%) of the Gross Domestic Product (GDP) and the GDP per capita (current US$).^2^^,^^41^ We included the CHE in absolute terms. In this case, for the interpretation of the CHE measure we consider 1000 dollars, representing how much an increase of 1000 USD would increase the outcome.

GII: this variable relates to reproductive health (maternal mortality ratio and adolescent fertility), empowerment (share of parliament seat and secondary/higher education attainment) and labour market (participation in the workforce) dimensions.^42^ The GII varies from 0 to 1, where 0 (the best scenario) indicates that women and men fare equally, and 1 (the worst scenario) means that men or women fare poorly compared to each other in all dimensions.

More detailed information on independent factors definition and classification is available in Table 1.

**Table 1:** Independent factors (individual- and country-level factors), definition and classification.

Independent factors	Definition	Classification
**Individual-level**		
Marital status	Marital status	Unmarried sexually active
		Married/in a union
Woman's age	Current woman's age in years	15–19
		20–34
		35–49
Woman's education	Woman's schooling level	None
		Primary/elementary school
		Secondary
		Higher
Wealth index	Wealth index constructed based on a principal component analysis including household characteristics and ownership of selected assets,^40^ dividing the score into quintiles, the first quintile represented the poorest 20%	Poorest
		2nd
		3rd
		4th
		Wealthiest
Area of residence	Area of residence	Urban
		Rural
Number of children	Number of children currently alive for each woman	0
		1
		2
		3 or more
**Country-level**		
CHE	The current health expenditure (CHE) was measured by multiplying the CHE as a percentage (%) of the Gross Domestic Product (GDP), adjusted for purchasing power parity. We included the CHE in absolute terms. For interpretation purposes, in the CHE we consider not one, but 1000 dollars. The CHE%GDP data were accessed in the Global Health Expenditure Database <https://apps.who.int/nha/database/Select/Indicators/en>. Data for GDP per capita were available at the World Bank website data <https://data.worldbank.org/indicator/NY.GDP.PCAP.CD?view=chart>.	CHE in dollar value.
GII	GII: this variable relates to reproductive health, empowerment and labour market dimensions. The GII varies from 0 to 1, where 0 (the best scenario) indicates that women and men fare equally, and 1 (the worst scenario) means that men or women fare poorly compared to each other in all dimensions. In this study, we divided the GII in tertiles, where (1st: GII = [0.291, 0.433], 2nd: GII = [0.449, 0.477], and 3rd: GII = [0.479, 0.776]), and the 1st tertile representing the most equitable group of countries and the 3rd the most unequal countries. For the Gender Inequality Index (GII) data were available at Human Development Data Center <http://hdr.undp.org/en/data>	Least
		2nd
		Highest

### Statistical analyses

Descriptive analyses were performed to present the distribution of individual and contextual factors. DFPSm coverage and 95% confidence intervals (95% CI) for each country were described, and the median coverage for all LAC countries included. We also presented relative frequencies of the individual-level factors and described the DFPSm coverage and 95% CI according to these characteristics.

Crude and adjusted multilevel logistic regression with two levels were conducted: individuals (level 1) and country (level 2). Odds ratios (OR) and the corresponding 95% CIs were calculated, and the intraclass correlation coefficient (ICC) was estimated. The ICC varies from 0 to 1 and quantifies the proportion of the observed variability of the outcome that is explained by the country effect.^43^ In other words, the ICC in this study represents the percentage of the DFPSm variability explained by the difference between countries (level 2).

We performed the null model, a multilevel model with no predictor, allowing us to assess whether there was significant between-country variation in the DFPSm pattern. The unadjusted model, a single-level univariate model, was also estimated. In addition, we performed three other models. Model 1 was fitted only with individual-level factors. Model 2 was fitted only with country-level factors, and Model 3 was fitted with individual- and country-level factors. We used the Akaike information criterion (AIC) and Bayesian information criterion (BIC) to assess the model's goodness of fit. Smaller values of AIC and BIC represent better fitted models.

Statistical analyses were conducted using Stata 17.0 software (StataCorp LLC, College Station, TX, USA) and accounted for survey sample weights.

### Role of the funding source

This work was supported by the Bill and Melinda Gates Foundation [OPP1199234] and [INV-010051]. The Associação Brasileira de Saúde Coletiva (ABRASCO) also acts as a sponsor of the study. The funders had no role in study design, data collection and analysis, decision to publish, or preparation of the manuscript.

## Results

We included data of 109,149 sexually active women (unweighted number) from 14 LAC countries (Table 2). Women included in the analyses were mainly married/in a union (85.0%), aged 20–34 years (52.2%), from secondary or higher educational levels (66.6%), and residents in urban areas (64.0%). Around 65% of the women had two or more children (Table 3).

**Table 2:** Overall description of the 14 Latin American and Caribbean countries, survey characteristics, and the sample included in this study.

Country	Survey year	Survey type	Income level	Female population aged 15–49	CHE (thousands)	GII	Number of women^a^
Belize	2015	MICS	Upper-middle	99,237	28.3	0.423	2549
Colombia	2015	DHS	Upper-middle	12,952,328	46.5	0.433	21,551
Costa Rica	2018	MICS	Upper-middle	1,309,299	91.1	0.291	4286
Cuba	2019	MICS	Upper-middle	2,574,011	101.2	0.304	6448
Dominican Republic	2014	MICS	Upper-middle	2,677,150	38.0	0.477	17,040
El Salvador	2014	MICS	Lower-middle	1,764,001	27.6	0.400	7127
Guatemala	2014	DHS	Lower-middle	4,167,459	22.3	0.511	11,719
Guyana	2014	MICS	Lower-middle	199,834	21.3	0.479	2825
Haiti	2016	DHS	Low	2,874,391	6.3	0.776	6521
Honduras	2011	DHS	Lower-middle	2,196,087	18.2	0.469	11,766
Mexico	2015	MICS	Upper-middle	33,378,761	55.0	0.347	7287
Paraguay	2016	MICS	Upper-middle	1,766,326	35.6	0.452	4485
Suriname	2018	MICS	Upper-middle	146,653	54.8	0.449	3862
Trinidad and Tobago	2011	MICS	High	364,206	89.9	0.359	1683

CHE = (GDP ∗ CHE%GDP), where: CHE–Current Health Expenditure; GDP–Gross Domestic Product per capita (current US$); CHE % GDP–CHE as percentage (%) of GDP.

GII–Gender Inequality Index.

a Unweighted number of sexually active women analysed in each survey.

**Table 3:** Individual-level factors and coverage (95% Confidence Interval, CI) of demand for family planning satisfied by modern contraceptive methods (DFPSm).

Individual-level factors	Distribution (%)	DFPSm % (95% CI)
Marital status		
Unmarried sexually active	15.0	63.1 (55.1–71.2)
Married/in a union	85.0	71.3 (63.8–78.9)
Woman's age (years)		
15–19	7.6	55.6 (45.9–65.4)
20–34	52.2	69.7 (62.8–76.7)
35–49	40.2	73.6 (65.8–81.4)
Woman's education		
None	5.1	63.8 (55.6–72.0)
Primary/elementary school	28.3	69.9 (61.9–77.8)
Secondary	46.5	70.4 (62.9–78.0)
Higher	20.1	71.9 (65.4–78.4)
Wealth index		
Poorest	17.9	62.8 (53.5–72.2)
2nd	19.6	68.6 (60.4–76.7)
3rd	20.7	71.2 (64.1–78.3)
4th	21.3	72.7 (65.3–80.2)
Wealthiest	20.5	74.3 (66.8–81.8)
Area of residence		
Urban	64.0	71.8 (64.6–79.0)
Rural	36.0	67.9 (60.1–75.8)
Number of children		
0	12.5	56.3 (47.4–65.2)
1	22.3	68.6 (61.6–75.5)
2	27.9	74.0 (67.4–80.5)
3 or more	37.3	73.9 (65.5–82.3)

All countries, except for Haiti (low-income) and Trinidad and Tobago (high-income), were from lower- or upper-middle-income levels (Table 2). Mexico has the largest female population aged 15–49 (33,378,761), followed by Colombia (12,952,328), while Belize (99,237) was the country with the smallest female population.

Regarding the contextual factor's description, Cuba (101,160), Costa Rica (91,090), and Trinidad and Tobago (89,879) presented the highest CHE, while Haiti had the lowest value (6,323) (Table 2). Regarding the GII, Haiti shows the worst scenario in terms of gender inequality, showing that men or women fare poorly compared to each other in all dimensions (GII: 0.776), while Costa Rica presented the best scenario (GII: 0.291), indicating that in this country women and men fare more equally.

**Fig. 1:**
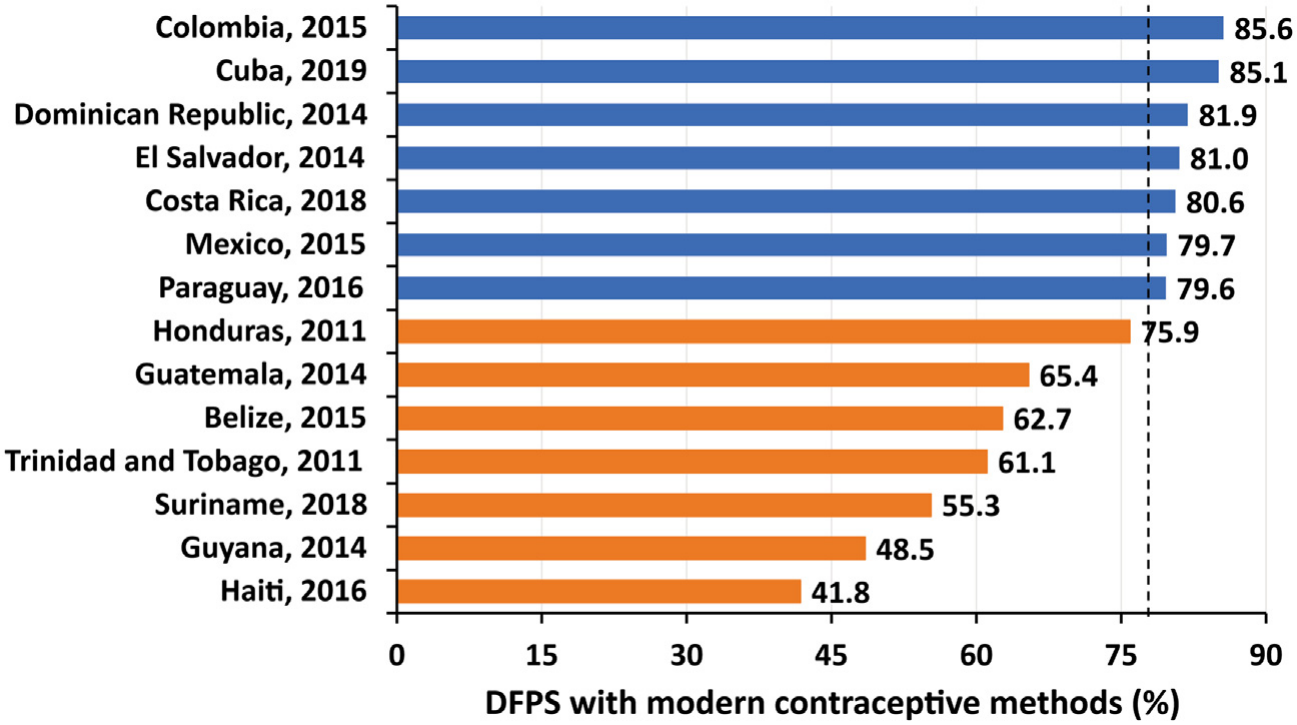
Coverage of demand for family planning satisfied by modern contraceptive methods (DFPSm) (%) in 14 Latin American and Caribbean countries. The orange color indicates below median value (77.8), and the blue color represents above median value (77.8).

The coverage of DFPSm in the 14 Latin American and Caribbean countries included in the analyses ranged from 41.8% (95% CI: 40.2–43.5) in Haiti to 85.6% (95% CI: 84.9–86.3) in Colombia, with an overall median coverage of 77.8% (Fig. 1). Trinidad and Tobago, the only high-income country, presented coverage almost 17 percentage points below the median value. Although there were no coverage differences between Colombia and Cuba, these countries have shown a significantly higher coverage compared to the other ones—14.9 and 14.4 percentage points higher, respectively, compared to the median coverage of other countries. On the other hand, Haiti has presented significantly lower DFPSm coverage than all other LAC countries analysed (Fig. 1). Among the individual-level subgroups, married women or in a union, aged 35–49 years, with higher education, the wealthiest, living in urban areas, with more than one child presented the highest coverages of DFPSm (Table 3).

The null model ICC showed a value of 11.8%, indicating that 11.8% of the variation in DFPSm was attributable to differences across the countries (Table 4). Table 4 also showed the unadjusted effects of the individual and country-level variables on DFPSm derived from the multilevel logistic regression model. According to the results, married and educated women, older than 20 years old, in the highest level of wealth, from urban areas, and with more than one child are significantly more likely to present DFPSm.

**Table 4:** Results from multilevel logistic regression analysis investigating the association between individual- and country-level factors and demand for family planning satisfied by modern contraceptive methods (DFPSm) among women in Latin American and Caribbean countries.

Variables	Null model	Unadjusted model OR (95% CI)	Model 1 OR (95% CI)	Model 2 OR (95% CI)	Model 3 OR (95% CI)
**Individual-level**					
Marital status					
Unmarried sexually active		1	1		1
Married/in a union		**1.51 (1.28–1.77)**	1.04 (0.85–1.28)		1.05 (0.85–1.28)
Woman's age (years)					
15–19		1	1		1
20–34		**1.95 (1.68–2.26)**	**1.27 (1.07–1.50)**		**1.26 (1.07–1.50)**
35–49		**2.39 (1.98–2.89)**	**1.27 (1.07–1.51)**		**1.26 (1.06–1.50)**
Woman's education					
None		1	1		1
Primary/elementary school		**1.35 (1.12–1.63)**	**1.32 (1.12–1.55)**		**1.31 (1.11–1.54)**
Secondary		**1.39 (1.06–1.81)**	**1.43 (1.16–1.76)**		**1.42 (1.16–1.74)**
Higher		**1.50 (1.13–1.99)**	**1.53 (1.28–1.82)**		**1.51 (1.27–1.81)**
Wealth index					
Poorest		1	1		1
2nd		**1.32 (1.21–1.45)**	**1.33 (1.23–1.43)**		**1.33 (1.23–1.43)**
3rd		**1.51 (1.26–1.81)**	**1.51 (1.31–1.74)**		**1.51 (1.31–1.75)**
4th		**1.65 (1.25–2.17)**	**1.65 (1.32–2.06)**		**1.65 (1.32–2.07)**
Wealthiest		**1.80 (1.27–2.55)**	**1.80 (1.35–2.39)**		**1.81 (1.36–2.41)**
Area of residence					
Urban		1	1		1
Rural		**0.82 (0.68–0.99)**	0.95 (0.89–1.03)		0.96 (0.89–1.03)
Number of children					
0		1	1		1
1		**1.79 (1.51–2.11)**	**1.78 (1.43–2.23)**		**1.79 (1.43–2.24)**
2		**2.38 (1.88–3.02)**	**2.35 (1.72–3.21)**		**2.35 (1.72–3.22)**
3 or more		**2.37 (1.79–3.14)**	**2.65 (1.82–3.85)**		**2.66 (1.82–3.88)**
**Country-level**					
CHE		1.01 (1.00–1.02)		1.00 (0.99–1.01)	1.00 (0.99–1.02)
GII					
Least		1		1	1
2nd		0.83 (0.43–1.62)		0.83 (0.37–1.88)	0.83 (0.37–1.86)
Highest		**0.31 (0.17–0.59)**		**0.32 (0.13–0.76)**	**0.32 (0.14–0.74)**
**ICC (%)**	11.8		12.1	6.6	6.6
**Model goodness of fit**					
Log pseudolikelihood	−5677		−5525	−5672	−5521
AIC	11,357		11,076	11,354	11,067
BIC	11,376		11,201	11,402	11,192
N^a^	109,103		109,103	109,103	109,103

Null model: a multilevel model with no predictor. Unadjusted model: a single-level univariate model. Model 1: a single-level multivariate adjusted model. Model 2: a multilevel-model with only country-level predictors. Model 3: the fully-adjusted multi-level model.

CHE = (GDP ∗ CHE%GDP), where: CHE–Current Health Expenditure; GDP–Gross Domestic Product per capita (current US$); CHE % GDP–CHE as percentage (%) of GDP.

GII–Gender Inequality Index; OR–Odds ratios; CI–Confidence Interval.

Bold letter indicate p < 0.05.

a Unweighted sample size.

About the country-level crude effects, we found that women living in high gender inequality countries significantly have an odd of DFPSm 70% lower compared to those living in countries with low gender inequality. CHE level of countries was not associated with DFPSm (Table 4).

Adjusted results from Model 1, which only includes individual-level variables, showed that the significant effect of woman's age and education, wealth index, and the number of children remained after adjustment. Women aged 20–34 and those 35–49 had 27% higher odds of having DFPSm. Women with three or more children had 2.65 (95% CI: 1.82–3.85) times higher odds of DFPSm than women without children. The association of marital status and area of residence with DFPSm disappeared after adjustment.

Model 2 in Table 4 shows the effect of the country-level variables where, again, the only variable associated with DFPSm was the GII, in which women with the highest GII tertile were less likely (OR: 0.32, 95% CI: 0.13–0.76) to have demand for family planning satisfied by modern contraceptive methods than those living in countries from the lowest GII tertile.

Results from Model 3, which include both individual and country-level predictors, show that the association of residing in countries with high gender inequalities was virtually unaltered by adjustment. After adjustment for individual-level variables, the odds of DFPSm for women from countries belonging to the highest tertile of GII remained lower when compared to those women from countries in the lowest tertile.

According to the AIC criterion, Model 3 fits better than the others. Finally, the results of the ICC of the adjusted models showed that individual and country-level variables contributed to explain the differences between countries. In the final model, the ICC was 6.6%, indicating that around 7% of the variation in DFPSm could still be attributable to differences across the countries.

## Discussion

To the best of our knowledge, this is the first multi-country research investigating the association between Gender Inequality and Health Expenditure with DFPSm in sexually active women from LAC countries. We found that the country-level factor GII, beyond individual-level factors, plays a relevant role in explaining the variations in the coverage of DFPSm in sexually active women from LAC countries. The coverage of DFPSm varied greatly among the LAC countries, with a median value of 77.8%, ranging from Haiti with the lowest median coverage of DFPSm to Colombia with the highest value. DFPSm was directly associated with woman's education, wealth index, and the number of children.

In the LAC region, many countries provide contraceptive methods free of charge in public facilities, despite the still present inequalities.^7^ Furthermore, family planning policies are directed to protect and promote women's rights, guarantee gender equality, and other important issues related to sexual and reproductive health.^44^ More detailed information on this topic and the respective search strategies are available in the Supplementary Material (Files 2, 3, and 4).

Among sexually active women from LAC countries, the coverage of DFPSm varied from 43.4% in Bolivia to 89.5% in Cuba, with coverage inequalities in which the poorest, youngest, less educated women and those living in rural areas presented the lowest DFPSm.^3^ Initiatives that may contribute to reducing inequalities are present in the LAC region, such as Conditional and Unconditional Cash Transfer Programs. However, findings about the direct impacts of these programs on the use of modern contraception are inconsistent.^45^ Improving individual-level factors may contribute to family planning advances beyond health and social changes.

The importance of country-level socioeconomic determinants is also mentioned in the scientific literature, despite the lack of consensus on which factors are more relevant for different health-related outcomes in each region and country.^19^^,^^46^^,^^47^ This study added that GII, a measure of disadvantages affecting women, which has empowerment, reproductive health, and labour market as its dimensions, plays a relevant role in the coverage of DFPSm.^48^ Regarding empowerment, a study found an association between different empowerment dimensions and the use of contraception.^15^ Empowerment measures were developed, validated, and expanded. It is currently possible to apply these measures to different low-and middle-income countries.^49^^,^^50^ Women's empowerment is frequently related to family planning and the possible reproductive health benefits for women.^20^^,^^21^ In addition, one possible hypothesis is that participation in the labour market and reproductive autonomy mediate the relationship between gender inequality and DFPSm. Furthermore, higher scores of GII were related to other health outcomes, such as lower life expectancy and healthy life expectancy, as well as increased years of life lost, morbidity and years lived with disability.^19^

CHE is a complex measure included in this analysis. So, it is important to be cautious when interpreting its country-level effects. The result that only GII was significant does not mean that health investments are irrelevant to family planning. Besides, sexual and reproductive health investments may be shared between maternal health and family planning.

Although this study focused on GII and CHE, many other contextual factors are available on databases around the world, such as Gross National Income (GNI), poverty headcount ratio at national poverty lines, population density, and density of health centres, which are directly related in the other indicators or that there is a lack of data for many countries. In view of comparison purposes, investigating countries from other regions may also be important to understand the country-level variations in DFPSm worldwide. Sexual acceptability of contraception and its broad range of related macro, relational, and individual factors may contribute to contraceptive use, implying that family planning includes a range of factors that are not always captured in common models.^51^

One of the strengths of this study is including information for 14 LAC countries. In addition, the demand for family planning satisfied measure shows advantages over the measure of the prevalence of contraceptive use since it includes in its denominator only women who are in need of contraception.^3^^,^^30^^,^^52^ Many contraceptive methods are available for couples, and modern methods are more effective and less prone to failure for all age subgroups.^38^^,^^53^ Furthermore, modern contraceptive methods are key to preventing unplanned pregnancies, which may impact women, children, and families lives. Unplanned pregnancies are associated with adverse outcomes such as less education access for adolescents, unsafe abortion, late antenatal care, and its possible consequences on women's and child's health and increased health system expenditures.^54^^,^^55^

Although DFPSm presents the advantage of including women who need contraception in its denominator, there are limitations to this indicator. Problems are mainly related to the lack of information on the coital frequency, especially among married women (the subgroup on which most studies about modern contraception focus), the lack of information on the contextual scenario, and the type of contraception women have more access to in each country.^3^^,^^30^^,^^52^ This study did not include a factor about family planning availability, despite its possible influences on family planning outcomes. One limitation identified during the conduction of this study was the lack of contextual data availability on expenditure on reproductive health and family planning for LAC countries. In June 2022, when the final version of the data analysis was performed, only two LAC countries included in this analysis (Guyana and Haiti) presented data on current health expenditure on reproductive health and specifically on contraceptive management (family planning) available in the Global Health Expenditure Database (https://apps.who.int/nha/database/Select/Indicators/en). Regarding the description of the CHE results, the data order may vary according to the unit of measurement used.

Furthermore, efforts are needed to improve the current indicators by performing more contextualised modern contraception comparisons worldwide. One of the limitations of performing this type of analysis is that not all countries have data on family planning at the country level, making it difficult to carry out this type of study. Furthermore, this study included data from a pre-COVID-19 pandemic scenario. Currently, there is evidence pointing out that changes in family planning occurred, such as specific disruptions in contraceptive use, resulting in unintended pregnancies.^56^^,^^57^ The findings of this study need to be interpreted considering this new reality, given its possible implications, contributing to planning different strategies. Strengthening the databases with standardised country-level data on access to family planning, health care facilities, and family planning policies, among others, may facilitate future comparisons and possible generalisations for similar realities.

### Conclusion

The country-level factor GII, beyond individual-level factors, plays a relevant role in explaining the variations in the coverage of DFPSm in sexually active women from LAC countries. Less gender inequality at the country level may play a relevant role in the coverage of DFPSm. In addition, individual factors such as woman's age, education, wealth index, and the number of children also contribute to modern contraceptive coverage. In this sense, planning actions, including macro-level approaches focusing on reducing gender disparities and considering individual-level factors, may be essential to guarantee reproductive health to the population of women in need of contraception.

## Contributors

LRM conceived the idea of the present article, analysed and interpreted the data, and wrote the manuscript. CB, FE, and LPVR contributed to the conceptualisation, methodology, formal analysis, and substantial manuscript revision. BECV, AB, ARV, FH, RGPL, AJDB, and MFS substantially revised the manuscript and contributed to data interpretation. FCW conceived the idea of the present article, supervised the project, and participated in all subsequent steps. All authors commented on the draft manuscript and approved its final version. The authors do not report any conflicting interests.

## Data sharing statement

The data are anonymised and geographically scrambled to ensure confidentiality and are publicly available through the agencies’ websites.

## Declaration of interests

The authors declare no conflict of interest. RGPL, who is a staff member of the Pan American Health Organization, hold sole responsibility for the views expressed in their texts, which may not necessarily reflect the opinion or policy of the Pan American Health Organization.
