# Supplementary material for: The role of gender inequality and health expenditure on the coverage of demand for family planning satisfied by modern contraceptives: a multilevel analysis of cross-sectional studies in 14 LAC countries
Source: Lancet Reg Health Am. 2023 Feb 8;19:100435. doi: 10.1016/j.lana.2023.100435 (PMC10025422; doi:10.1016/j.lana.2023.100435)
Supplement: Supplementary Material [file mmc1.docx]

**Supplementary Material**

**Table of Contents**

***File 1*: Demographic and Health Surveys and Multiple Indicator Cluster Surveys explanation.**

***File 2*: Search strategies at Scopus, Web of Science, Pubmed and LILACS databases, and other sources according to Latin America and the Caribbean Region and each country studied.**

***File 3*: Additional general and government search sources.**

***File 4*: Family planning policies, contraception availability, and additional information on sexual and reproductive health by country**

***File 1*: Demographic and Health Surveys and Multiple Indicator Cluster Surveys explanation.**

Demographic and Health Surveys

The Demographic and Health Surveys (DHS) were developed by USAID in partnership with countries to provide reliable information for monitoring a wide range of health indicators. It started in the 80s to provide a broad picture of women’s and children's health. Standard DHS surveys are nationally representative surveys, with sample sizes ranging from 5,000 to 30,000 households.

Multiple Indicator Cluster Surveys

Unicef started in the mid-1990’s the Multiple Indicator Clusters Survey (MICS). As of November 2022, 349 surveys were conducted in 118 countries. These surveys aim to provide statistically sound and internationally comparable estimates of children's and women's health indicators. The Unicef headquarters plan the survey with the country’s office and the support of the governments. During the planning of the surveys, the indicators gaps are discussed, and the survey design is developed. Households are sampled to produce nationally representative data.

Similarities between DHS and MICS surveys

DHS and MICS surveys used face-to-face interviews, with standardized questionnaires applied to women aged 15-49 years old. All interviewed women are household *de jure* members. The topics covered by both surveys include several phases of the continuum of care of reproductive, maternal and child health: family planning, antenatal care and delivery, child immunization, childhood disease management, nutrition, and several other indicators.

These surveys consisted of a very useful tool for conducting studies on family planning. A search in Pubmed with the terms (DHS and “Family planning”) resulted in 254 references found, while (MICS and “Family planning”) resulted in 40 references found. Most of these references were African or multi-country studies.

With the family planning module, several indicators related to knowledge and use of contraceptives can be extracted. Also, it is possible to define those in need of contraception. A woman was considered in need of contraception if she was sexually active, fecund and did not want to become pregnant within the next 2 years, or if she was unsure about whether or when she wanted to become pregnant. Pregnant women with a mistimed or unintended pregnancy are also considered in need of contraception. There is a slight difference in MICS and DHS surveys related to the denominator definition of the indicator. When re-analyzing surveys, DHS provides a variable of “need of contraception” while in MICS, each component of “in need of contraception” is calculated separately. According to the DHS reports, this variable of need of contraception was constructed considering the same characteristics, specifically: women who were not pregnant and not postpartum amenorrhoeic and were considered fecund and want to postpone their next birth for 2 or more years or stop childbearing altogether but are not using a contraceptive method; women who had a mistimed or unwanted current pregnancy; or women who were postpartum amenorrhoeic and her most recent birth in the last 2 years was mistimed or unwanted. Although this slight difference in estimates of both surveys, they are comparable and used in extensive multi-countries/surveys studies.^1-6^

**References (File 1):**

1. Barros AJD, Boerma T, Hosseinpoor AR, Restrepo-Méndez MC, Wong KLM, Victora CG. Estimating family planning coverage from contraceptive prevalence using national household surveys. *Glob Health Action*. 2015 Dec 9;8(1):29735. Available from: https://doi.org/10.3402/gha.v8.29735

2. Costa JC, Mujica OJ, Gatica-Domínguez G, del Pino S, Carvajal L, Sanhueza A, et al. Inequalities in the health, nutrition, and wellbeing of Afrodescendant women and children: A cross-sectional analysis of ten Latin American and Caribbean countries. *Lancet Reg Heal*. 2022;15:100345. Available from: https://doi.org/10.1016/j.lana.2022.100345

3. Ewerling F, Victora CG, Raj A, Coll CVN, Hellwig F, Barros AJD. Demand for family planning satisfied with modern methods among sexually active women in low- and middle-income countries: who is lagging behind? *Reprod Health*. 2018 Dec 6;15(1):42. Available from: https://doi.org/10.1186/s12978-018-0483-x

4. Haakenstad A, Angelino O, Irvine CMS, Bhutta ZA, Bienhoff K, Bintz C, et al. Measuring contraceptive method mix, prevalence, and demand satisfied by age and marital status in 204 countries and territories, 1970–2019: a systematic analysis for the Global Burden of Disease Study 2019. *Lancet*. 2022;400(10348):295–327. Available from: https://doi.org/10.1016/S0140-6736(22)00936-9

5. Mesenburg MA, Restrepo-Mendez MC, Amigo H, Balandrán AD, Barbosa-Verdun MA, Caicedo-Velásquez B, et al. Ethnic group inequalities in coverage with reproductive, maternal and child health interventions: cross-sectional analyses of national surveys in 16 Latin American and Caribbean countries. *Lancet Glob Heal*. 2018;6(8):e902–13. Available from: https://doi.org/10.1016/S2214-109X(18)30300-0

6. Ponce de Leon RG, Ewerling F, Serruya SJ, Silveira MF, Sanhueza A, Moazzam A, et al. Contraceptive use in Latin America and the Caribbean with a focus on long-acting reversible contraceptives: prevalence and inequalities in 23 countries. *Lancet Glob Heal*. 2019 Feb;7(2):e227–35. Available from: https://doi.org/10.1016/S2214-109X(18)30481-9

***File 2*: Search strategies at Scopus, Web of Science, Pubmed and LILACS databases, and other sources according to Latin America and the Caribbean Region and each country studied.**

| **Overall** | **Database, Search Date, and** **Search Strategies** | | | |
| --- | --- | --- | --- | --- |
| **Region/ Country** | **Scopus**  **2021/12/21** | **Web of Science**  **2021/12/20** | **Pubmed**  **2021/12/20** | **LILACS**  **2021/12/16** |
| Latin America and the Caribbean Region | (“Family Planning”) AND ((“Latin America”) OR (“Caribbean Region”))  Titles: 1746  (“Family Planning Policy”) AND ((“Latin America”) OR (“Caribbean Region”))  Titles: 126  Restriction:  * **Article title, Abstract and Keywords** | (“Family Planning”) AND ((Latin America) OR (Caribbean Region))  Titles: 190  (“Family Planning Policy”) AND ((Latin America) OR (Caribbean Region))  Titles: 0  Restriction:  ***** **All Fields** | (“Family Planning”) AND ((Latin America) OR (Caribbean Region))  Titles: 2011  (“Family Planning Policy”) AND ((Latin America) OR (Caribbean Region))  Titles: 124  Restriction:  ***** **All Fields** | (Family Planning)  Titles: 583  (Family Planning Policy)  Titles: 61  <https://lilacs.bvsalud.org/en/>  Restriction:  * **No campo: Palavras** |
| Belize | (“Family Planning”) AND (“Belize”)  Titles: 19 | (“Family Planning”) AND (“Belize”)  Titles: 6 | (“Family Planning”) AND (“Belize”)  Titles: 17 |  |
| Colombia | (“Family Planning”) AND (“Colombia”)  Titles: 381 | (“Family Planning”) AND (“Colombia”)  Titles: 110 | (“Family Planning”) AND (“Colombia”)  Titles: 359 |  |
| Costa Rica | (“Family Planning”) AND (“Costa Rica”)  Titles: 125 | (“Family Planning”) AND (“Costa Rica”)  Titles: 35 | (“Family Planning”) AND (“Costa Rica”)  Titles: 117 |  |
| Cuba | (“Family Planning”) AND (“Cuba”)  Titles: 102 | (“Family Planning”) AND (“Cuba”)  Titles: 41 | (“Family Planning”) AND (“Cuba”)  Titles: 99 |  |
| Dominican Republic | (“Family Planning”) AND (“Dominican Republic”)  Titles: 156 | (“Family Planning”) AND (“Dominican Republic”)  Titles: 40 | (“Family Planning”) AND (“Dominican Republic”)  Titles: 160 |  |
| El Salvador | (“Family Planning”) AND (“El Salvador”)  Titles: 70 | (“Family Planning”) AND (“El Salvador”)  Titles: 16 | (“Family Planning”) AND (“El Salvador”)  Titles: 81 |  |
| Guatemala | (“Family Planning”) AND (“Guatemala”)  Titles: 172 | (“Family Planning”) AND (“Guatemala”)  Titles: 86 | (“Family Planning”) AND (“Guatemala”)  Titles: 148 |  |
| Guyana | (“Family Planning”) AND (“Guyana”)  Titles: 17 | (“Family Planning”) AND (“Guyana”)  Titles: 3 | (“Family Planning”) AND (“Guyana”)  Titles: 15 |  |
| Haiti | (“Family Planning”) AND (“Haiti”)  Titles: 119 | (“Family Planning”) AND (“Haiti”)  Titles: 52 | (“Family Planning”) AND (“Haiti”)  Titles: 107 |  |
| Honduras | (“Family Planning”) AND (“Honduras”)  Titles: 82 | (“Family Planning”) AND (“Honduras”)  Titles: 28 | (“Family Planning”) AND (“Honduras”)  Titles: 79 |  |
| Mexico | (“Family Planning”) AND (“Mexico”)  Titles: 919 | (“Family Planning”) AND (“Mexico”)  Titles: 431 | (“Family Planning”) AND (“Mexico”)  Titles: 952 |  |
| Paraguay | (“Family Planning”) AND (“Paraguay”)  Titles: 40 | (“Family Planning”) AND (“Paraguay”)  Titles: 15 | (“Family Planning”) AND (“Paraguay”)  Titles: 37 |  |
| Suriname | (“Family Planning”) AND (“Suriname”)  Titles: 9 | (“Family Planning”) AND (“Suriname”)  Titles: 4 | (“Family Planning”) AND (“Suriname”)  Titles: 8 |  |
| Trinidad and Tobago | (“Family Planning”) AND (“Trinidad and Tobago”)  Titles: 27 | (“Family Planning”) AND (“Trinidad and Tobago”)  Titles: 5 | (“Family Planning”) AND (“Trinidad and Tobago”)  Titles: 26 |  |
| **Total Titles** | **4110** | **1062** | **4340** | **644** |
|  | | | | |
| **Total Titles After Removing Duplicates** | **4197** | | | |
| **Titles Selected** | **673** | | | |
| **Abstracts Selected** | **85** | | | |
| **Articles**  **Selected** | **39** | | | |

***File 3*: Additional general and government search sources.**

| **Additional Sources** |
| --- |
| **General Sources (all countries)** |
| Constitute Project  <https://www.constituteproject.org>  Global Gender Equality Constitutional Database  <https://constitutions.unwomen.org/en>  Global Health Expenditure Database  <https://apps.who.int/nha/database/Select/Indicators/en>  PAHO  <https://www.paho.org/en>  USAID  <https://www.usaid.gov/>  WHO database  <https://www.who.int/news-room/fact-sheets/detail/adolescent-pregnancy>  World Bank Open Data  <https://data.worldbank.org/> |

***File 4*: Family planning policies, contraception availability, and additional information on sexual and reproductive health by country****.**

| **Country** | **Family Planning Policies and**  **Women’s Rights** | **Contraception Availability** | **Additional Information on Sexual and Reproductive Health** |
| --- | --- | --- | --- |
| 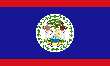  Belize | ^1^ Constitution: Policies of state protect the rights of the individual to basic health, ensure gender equality, eliminate disparities.  Other official documents related to women’s rights protection:  - Sexual and Reproductive Health Policy (2002)  - National Gender Policy (2002)  - Revised National Gender Policy (2013) | ^22^ Female sterilization, pill and injectables are modern contraceptive methods commonly used.  ^22^ Adolescents are less likely to use contraception and women with no education present the lowest percentages of use. | Total fertility rate in the survey year (2015): 2.439 births per woman^39^  Additional documents:  - National Strategic Plan for the Sexual and Reproductive Health Policy Implementation, Belize 2006-2010 (Ministry of Health)  - Horizon 2030 (Belize’s framework for National Development) |
| 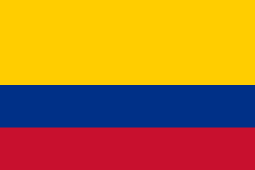  Colombia | ^2^ Constitution: “All individuals are guaranteed access to services that promote, protect, and restore health.” (Article 49). “The couple has the right to decide freely and responsibly the number of their children and shall support them and educate them while they are minors or non-self-supporting.” (Article 42)  ^3^ Other official documents:  - National Policy for Gender Equality  for Women and indicative Action Plan 2013-2016 | ^23,24^ Free of charge in public facilities as well as in contributory and subsidized regimes. Contraceptive methods available: Condom, injectable, implant, IUD, pill, sterilization (vasectomy, female sterilization).  ^25^ Difficulties to provide a wide range of contraceptive methods in all localities sometimes are present. | Total fertility rate in the survey year (2015): 1.863 births per woman^39^  ^25^ The public sector has a key role in the healthcare provision. The private sector also has participation in the family planning provision.  The PROFAMILIA Program (non-governmental organization) started in 1966, being important to guarantee sexual and reproductive health. More information at  <<https://profamilia.org.co/>> |
| 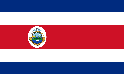  Costa Rica | ^4^ Constitution: “All persons have the right to a healthy and ecologically balanced environment.” (Article 50). “Consumers and users have the right to the protection of their health […]” (Article 46).  ^5^ All persons have the right to obtain adequate information and instructions on family planning (General Law of Health, Article 10).  ^3^ Another official document:  -National Policy on Gender Equality  and Equity 2007-2017 | ^24^ Condom, injectable, IUD, pill, sterilization are free of charge through Caja Costarricense de Seguridad Social (CCSS) - the national social health insurance scheme. Uninsured people must pay out of pocket.  ^26^ Indigenous and poor women, for example, face difficulty to access family planning. | Total fertility rate in the survey year (2018): 1.754 births per woman^39^  ^24^ There are very few uninsured in this country.  ^9^ Promotion of women’s rights:  - Law for the Promotion of Women's Social  Equality (created in the 1990s)  - National Institute of Women (INAMU). More information at  <<https://www.inamu.go.cr/web/inamu/inicio>> |
| 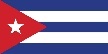  Cuba | ^6^ Constitution: Women and men have the same rights, responsibilities, opportunities, and possibilities. The State protects women from gender-based violence and ensures the exercise of their sexual and reproductive rights. (Article 43, Cuba 2019)  ^7^ Contraceptive methods are provided by the State. | ^7^ Free of charge. Contraceptive methods available: intrauterine devices (IUDs), condoms, injectables, and oral contraceptives. Limited availability of some contraceptive methods such as condoms in some localities. | Total fertility rate in the survey year (2019): 1.602 births per woman^39^  ^7^ Awareness-raising programs are important to the sexual and reproductive health services. Government institutions have a fundamental role in these issues. |
| 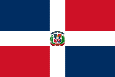  Dominican Republic | ^8^ Constitution: “All persons have the right to integral health.” (Article 61).  ^9^ Another official document:  - National Gender Equality and Equity Plan PLANEG II (2007-2017) | ^24^ Free of charge in public facilities as well as through Seguro Nacional de Salud (SeNaSa) and other public schemes. Contraceptive methods available: Condom, injectable, implant, IUD, pill, sterilization  ^27^ Difficulties to provide a wide range of contraceptive methods in all localities and access barriers for uninsured women in the lowest income quintiles sometimes are identified. | Total fertility rate in the survey year (2014): 2.423 births per woman^39^  ^27^  Bárcena A, editor. Gender equality plans in Latin America and the Caribbean: Road maps for development The modern contraceptive prevalence rate achieved a Plateauing, although differences in subgroups were identified.  Promotion of women’s rights:  - Ministry of Women (MMUJER). More information is available at <<https://mujer.gob.do/>> |
| 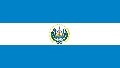  El Salvador | ^10^ Constitution: The health of the inhabitants is a State obligation and considered a public good. “The State shall determine the national health policy and shall control and supervise its application.” (Article 65).  ^11^ - Política de Salud Sexual y Reproductiva – important to advance in topics such as gender equity and women rights.  ^3^ - National Women’s Policy 2011-2014 | ^28^ Female sterilization is a modern contraceptive method commonly used. | Total fertility rate in the survey year (2014): 2.126 births per woman^39^  ^9^ Promotion of women’s rights:  - Salvadoran Institute for the Development of Women ISDEMU, created in 1996 <<https://isdemu.gob.sv/>>  - Law on Equality, Equity, and Non-discrimination against women.  - National Equality Plan 2016-2020 |
| 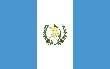  Guatemala | ^12^ Constitution: The health of the inhabitants is a State obligation and considered a public asset. “All persons and institutions are obligated to see to its conservation and reestablishment.” (Articles 94 - 95). The persons have the right to decide freely the number and the spacing of their children. (Article 47)  ^9^ Other official documents:  - Law on Dignity and Integral Promotion of Women  - National Policy for the Promotion and Integral Development of Women (PNPDIM)  - Opportunity Equity Plan 2008-2023 | ^24^ Free of charge public provision, with method availability varying according to the facility level. In general, the contraceptive methods condom, injectable, implant, IUD, pill, and sterilization are available. Although sterilization is free, there is limited availability. Injectable, IUD, pill, and sterilization (with limited availability) are free of charge through Instituto Guatemalteco de Seguridad Social (IGSS), a national social health insurance scheme, including postpartum sterilization. However, there are access difficulties for people who live in rural areas, indigenous and young women.^29^ | Total fertility rate in the survey year (2014): 3.088 births per woman^39^  ^29^ Health services concentration is in urban areas. The public sector has a key role in healthcare provision. However, due to limited availability in the public sector, some people seek the private sector for these services.  Promotion of women’s rights:  - Presidential Secretariat for Women (SEPREM). More information is available at  <<https://seprem.gob.gt/>> |
| 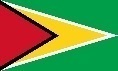  Guyana | ^13^ Constitution: There is a “Women and Gender Equality Commission” with specific women’s protection functions, such as to promote empowerment of women, women’s rights, scientific knowledge on women and gender with topics which includes reproductive health. (Article 212R). “Every citizen has a duty to participate in activities designed to improve the environment and protect the health of the nation.” (Article 25) | ^30^ Type of contraceptive methods available in public health facilities: Barrier Contraceptive Methods, Progesterone, Combined Oestrogen/ Progesterone, Long Acting Reversible Contraceptives (LARC). | Total fertility rate in the survey year (2014): 2.558 births per woman^39^  - National Health Sector Strategy 2013-2020  - Strategic Plan |
| 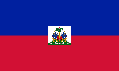  Haiti | ^14^ Constitution: All citizens have the right to health. “The State has the absolute obligation to guarantee the right to life, health, and respect of the human person for all citizens without distinction, in conformity with the Universal Declaration of the Rights of Man.” (Article 19). Equality of the sexes, equity of gender, and women’s political participation are guaranteed. | ^24^ There is limited availability of contraceptive methods such as condoms, injectables, implants, IUDs, pills, and sterilization, in public facilities. These contraceptive methods are free of charge through determined public schemes, although unavailability often occurs.  ^31^ Uninsured women face barriers to access family planning. | Total fertility rate in the survey year (2016): 3.041 births per woman^39^  ^24^ For this country, donations are important to guarantee family planning provision, and the private sector has a significant role.  ^31^ In 2010 occurred an earthquake, which affected many health aspects, including family planning provision. |
| 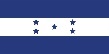  Honduras | ^15^ Constitution: “All men are born free and equal in rights”, being the discrimination of sex, for example, punishable (Article 60).  “[…]. The State shall maintain a satisfactory environment for the protection of everyone's health. […]” (Article 145).  ^9^ Other fundamental documents:  -National Policy on Women  -Second Gender Equality and Equity Plan (2010-2022) (II PIEGH) | ^24^ Free of charge in public facilities. Contraceptive methods available: Condom, injectable, implant, IUD, pill, sterilization  ^32^ Differences in subgroups according to the area of residence and wealth, for example, are identified (people living in rural areas and with the lowest income facing barriers to access family planning). | Total fertility rate in the survey year (2011): 2.86 births per woman^39^  ^32^ The private sector has significant participation in family planning supply.  ^9^ Promotion of women's rights:  - National Women's Institute INAM (Instituto Nacional de la Mujer). <<https://inam.gob.hn/index.php/mision-y-vision/>>  - Presidential Program "Ciudad Mujer" <<https://www.ciudadmujer.gob.hn/>> |
| 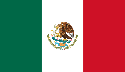  Mexico | ^16^ Constitution: “Man and woman are equal under the law. […] Every person has the right to decide, in a free, responsible, and informed manner, about the number of children desired and the timing between each of them. […]. Every person has the right to health protection.” (Article 4)  ^17^ All citizens have the right to family planning. | ^33^ Contraceptive methods available: IUD, implant, sterilization  ^33^ Health insurance: employment-based (*Seguro Social*); public insurance for those working in the informal sector, self-employed; otherwise without insurance (*Seguro Popular*); none. | Total fertility rate in the survey year (2015): 2.215 births per woman^39^  Promotion of women's rights:  - National Women's Institute (INMUJERES) <<https://www.gob.mx/inmujeres>>  - National Programme for Equal  Opportunities and Non-Discrimination  against Women, 2013-2018 |
| 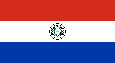  Paraguay | ^18^ Constitution: “The State recognizes the right of persons to freely and responsibly decide the number and the frequency of the birth of their children, as well as to receive, in coordination with the pertinent organs[,] education, scientific orientation, and adequate services in the matter. Special plans of reproductive health and maternal-child health [care] for people of scarce resources will be established.” (Article 61) | ^34, 35^ Contraceptive methods available: condom, pill, injectables, IUD, sterilization. | Total fertility rate in the survey year (2016): 2.479 births per woman^39^  Promotion of women's rights:  ^3^ - III National Plan for Equal  Opportunities for Women and Men,  2008-2017  - IV National Plan for Equal  Opportunities for Women and Men,  2018-2024 |
| 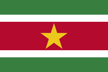  Suriname | ^19^ Constitution: “Everyone shall have a right to health. The State shall promote the general health care by systematic improvement of living and working conditions and shall give information on the protection of health.” (Article 36).  ^20^ The National Sexual and Reproductive  Health and Rights Policy of Suriname,  2020-2030 recognize the right to “universal access to sexual and reproductive health-care services, including family planning” (page 6). | ^36, 37^ Contraceptive methods available: Condom, pill, injectable, implant, sterilization. | Total fertility rate in the survey year (2018): 2.418 births per woman^39^  Promotion of women's rights:  ^40^- In Suriname 2017-2021 Policy Development Plan, family planning is cited as one of the important prevention issues that need knowledge and provision. |
| 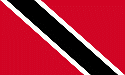  Trinidad  and  Tobago | ^21^ Constitution: The individual has fundamental human rights such as life, liberty, security  Other official documents:  - In the National Sexual and Reproductive Health Policy (2016), family planning is presented as one of the core points in Sexual and Reproductive Health services, being the access to all persons who need and require it fundamental.  ^3^ - National Policy on Gender and  Development of the Republic of Trinidad  and Tobago. Draft Document | ^38^ Contraceptive methods available: Pill, injection, condom, IUD (Copper), Sterilization. | Total fertility rate in the survey year (2011): 1.804 births per woman^39^  Promotion of women's rights:  ^38^ Family Planning Association of Trinidad and Tobago is important to sexual and reproductive health in this country. |

*This search was conducted in the Scopus, Web of Science, Pubmed, and LILACS databases. Websites from Latin America and the Caribbean organizations and government websites from the respective countries were also consulted to elaborate this Box. The main search strategies used are presented in the File 2 and File 3 of this Supplementary Material.

**References (File 4):**

^1^ Constitution of Belize (1981 rev 2011). Available at <https://www.constituteproject.org/constitution/Belize_2011?lang=en> Accessed 2022 Jan 21

^2^ Constitution of Colombia (1991 rev. 2015). Available at <https://www.constituteproject.org/constitution/Colombia_2015?lang=en> Accessed 2022 Jan 21

^3^ Bárcena A, editor. Gender equality plans in Latin America and the Caribbean: Road maps for development. *Comisión Económica para América Latina y el Caribe*; 2017. Available from: <https://www.cepal.org/sites/default/files/events/files/gender_equality_plans_in_latin_america_and_the_caribbean._road_maps_for_development.pdf>

^4^ Constitution of Costa Rica (1949 rev. 2020). Available at <https://www.constituteproject.org/constitution/Costa_Rica_2020?lang=en> Accessed 2022 Jan 21

^5^ General Law of Health (Law Nº 5395, rev. 2014). Available from: <https://www.ucr.ac.cr/medios/documentos/2015/LEY-5395.pdf>

^6^ Constitution of Cuba (2019). Available from: <https://www.constituteproject.org/constitution/Cuba_2019?lang=en> Accessed 2022 Jan 21

^7^ Samuels F, Santana AT, with Rocío FERNÁNDEZ, Valia SOLÍS, Georgia PLANK and Maria STAVROPOULOU. Family, sexuality, and sexual and reproductive health in Cuba. The role of social norms. Report. ODI. February 2020 Available from: <https://cdn.odi.org/media/documents/Family_sexuality_and_sexual_and_reproductive_health_in_Cuba_the_role_of_social_norms.pdf>

^8^ Constitution of Dominican Republic (2015). Available from: <https://www.constituteproject.org/constitution/Dominican_Republic_2015?lang=en> Accessed 2022 Jan 21

^9^ Council of Ministers of Women of Central America and the Dominican Republic (COMMCA). Regional Policy on Equity and Gender Equality of the Central American Integration System (SICA). *PRIEG/SICA 2014-2025*. First edition: December 2013. Second edition (first update): March 2018

^10^ Constitution of El Salvador 1983 (rev. 2014). Available from: <https://www.constituteproject.org/constitution/El_Salvador_2014?lang=en> Accessed 2022 Jan 21

^11^ Ministerio de Salud (MOH). *Política de Salud Sexual y Reproductiva.* San Salvador, El Salvador: MOH; 2012. Available from: <https://www.salud.gob.sv/servicios/descargas/documentos/Documentaci%C3%B3n-Institucional/Pol%C3%ADtica-de-Salud-Sexual-y-Reproductiva/Politica-de-Salud-Sexual-y-Reproductiva/>.

^12^ Constitution of Guatemala 1985 (rev. 1993). Available from: <https://www.constituteproject.org/constitution/Guatemala_1993?lang=en> Accessed 2022 Jan 21

^13^ Constitution of Guyana 1980 (rev. 2016). Available from: <https://www.constituteproject.org/constitution/Guyana_2016?lang=en> Accessed 2022 Jan 21

^14^ Constitution of Haiti 1987 (rev. 2012). Available from: <https://www.constituteproject.org/constitution/Haiti_2012?lang=en> Accessed 2022 Jan 21

^15^ Constitution of Honduras 1982 (rev. 2013). Available from: <https://www.constituteproject.org/constitution/Honduras_2013?lang=en> Accessed 2022 Jan 21

^16^ Constitution of Mexico 1917 (rev. 2015). Available from: <https://www.constituteproject.org/constitution/Mexico_2015?lang=en> Accessed 2022 Jan 21

^17^ Camara de Diputados del H. Congreso de la Unión. (1984). Ley general de Salud. México, D.F.: Available from: <https://www.diputados.gob.mx/LeyesBiblio/pdf_mov/Ley_General_de_Salud.pdf>

^18^ Constitution of Paraguay (1992 rev 2011). Available from: <https://www.constituteproject.org/constitution/Paraguay_2011?lang=en>

Accessed 2022 Jan 21

^19^ Constitution of Suriname 1987 (rev. 1992). Available from: <https://www.constituteproject.org/constitution/Surinam_1992?lang=en> Accessed 2022 Jan 21

^20^ National Sexual and Reproductive Health and Rights Policy of Suriname, 2020-2030. Available from: <https://suriname.un.org/sites/default/files/2020-09/Final%20Suriname%20SRH%20policy%202020-2030%20J.Terborg_MOH_UNFPA2%2C%20JT290420.pdf>

^21^ Constitution of Trinidad and Tobago 1976 (rev. 2007). Available from: <https://www.constituteproject.org/constitution/Trinidad_and_Tobago_2007?lang=en> Accessed 2022 Jan 21

^22^ Statistical Institute of Belize and UNICEF Belize. 2017. Belize Multiple Indicator Cluster Survey, 2015-2016, Final Report. Belmopan, Belize: Statistical Institute of Belize and UNICEF Belize. Available from: <https://mics-surveys-prod.s3.amazonaws.com/MICS5/Latin%20America%20and%20Caribbean/Belize/2015-2016/Final/Belize%202015-16%20MICS_English.pdf>

^23^ Bertrand JT, Ross J, Sullivan TM, Hardee K, Shelton JD. Contraceptive method mix: updates and implications. *Global Health: Science and Practice*. 2020 Dec 23;8(4):666-79.

^24^ Fagan T, Dutta A, Rosen J, Olivetti A, Klein K. Family planning in the context of Latin America's universal health coverage agenda. *Global Health: Science and Practice*. 2017 Sep 27;5(3):382-98.

^25^ Health Policy Plus. 2016. Financing Family Planning: Colombia. Washington, DC: Palladium, *Health Policy Plus*.

^26^ Health Policy Plus. 2016. Financing Family Planning: Costa Rica. Washington, DC: Palladium, *Health Policy Plus*.

^27^ Health Policy Plus. 2016. Financing Family Planning: Dominican Republic. Washington, DC: Palladium, *Health Policy Plus*.

^28^ Mejía YM, Pérez CG. Contraceptive use in Latin America: knowledge of the population and influence of culture in the selection of a specific method. *Máster Universitario en Investigación en Ciencias Sociosanitarias*, 2022. Available at: <https://ebuah.uah.es/dspace/bitstream/handle/10017/53898/TFM_Mendez_Mejia_2022.pdf?sequence=1&isAllowed=y>

^29^ Health Policy Plus. 2016. Financing Family Planning: Guatemala. Washington, DC: Palladium, *Health Policy Plus*.

^30^ Singh P, Rozan R. TITLE: Essential Maternal Medicines - *Availability in hinterland Regions of Guyana*, 2019. Available from: <https://www.researchgate.net/profile/Rafi-Rozan-2/publication/363480879_TITLE_Essential_Maternal_Medicines_-Availability_in_hinterland_Regions_of_Guyana_2019_Researchers_Names/links/631f05a20a70852150eb2b1f/TITLE-Essential-Maternal-Medicines-Availability-in-hinterland-Regions-of-Guyana-2019-Researchers-Names.pdf>

^31^ Health Policy Plus. 2016. Financing Family Planning: Haiti. Washington, DC: Palladium, *Health Policy Plus*.

^32^ Health Policy Plus. 2016. Financing Family Planning: Honduras. Washington, DC: Palladium, *Health Policy Plus*.

^33^ Saavedra-Avendano B, Andrade-Romo Z, Rodriguez MI, Darney BG. Adolescents and long-acting reversible contraception: lessons from Mexico. *Maternal and child health journal*. 2017 Sep;21(9):1724-33.

^34^ Cordova-Pozo, Kathya, et al. "How do national contraception laws and policies address the contraceptive needs of adolescents in Paraguay?." *Reproductive health* 14.1 (2017): 1-9. Available from: <https://doi.org/10.1186/s12978-017-0344-z>

^35^ Santiso-Gálvez R, Ward VM, Bertrand JT. Family Planning in Paraguay. The Achievements of 50 Years. Chapel Hill, NC: MEASURE Evaluation; 2015. Available from: <https://www.measureevaluation.org/resources/publications/sr-15-118g/at_download/document>

^36^ National Sexual and Reproductive Health and Rights Policy of Suriname, 2020-2030. Available from: <https://suriname.un.org/sites/default/files/2020-09/Final%20Suriname%20SRH%20policy%202020-2030%20J.Terborg_MOH_UNFPA2%2C%20JT290420.pdf>

^37^ Ali M, Miller K, Gómez Ponce de Leon RF. Family planning and Zika virus: need for renewed and cohesive efforts to ensure availability of intrauterine contraception in Latin America and the Caribbean. *The European Journal of Contraception & Reproductive Health Care*. 2017 Mar 4;22(2):102-6.

^38^ Family Planning Association of Trinidad and Tobago. Available from: <http://www.ttfpa.org/>

^39^ The World Bank Group. Fertility rate, total (births per woman). Available from: <https://data.worldbank.org/indicator/SP.DYN.TFRT.IN>

^40^ Suriname Planning Bureau Foundation. 2017-2021 Policy Development Plan. January 2017. Available from: [https://suriname.un.org/sites/default/files/2020-10/2017-2021-DEVELOPMENT-PLAN.pdf Accessed 2022 Jan 31](https://suriname.un.org/sites/default/files/2020-10/2017-2021-DEVELOPMENT-PLAN.pdf%20Accessed%202022%20Jan%2031)
